# Maltol Promotes Mitophagy and Inhibits Oxidative Stress via the Nrf2/PINK1/Parkin Pathway after Spinal Cord Injury

**DOI:** 10.1155/2022/1337630

**Published:** 2022-02-01

**Authors:** Yuqin Mao, Jiqing Du, Xianghang Chen, Abdullah Al Mamun, Lin Cao, Yanhong Yang, Joana Mubwandarikwa, Muhammad Zaeem, Wanying Zhang, Yan Chen, Yusen Dai, Jian Xiao, Keyong Ye

**Affiliations:** Department of Orthopaedics, Affiliated Pingyang Hospital and School of Pharmaceutical Science, Wenzhou Medical University, Wenzhou, Zhejiang 325000, China

## Abstract

Spinal cord injury (SCI), a fatal disease in the central nervous system, is characteristic of weak neuronal regeneration ability and complex pathological progress. Activation of oxidative stress (OS) and apoptosis-mediated cell death significantly contributes to the progression of SCI. Current evidence suggests that maltol exerts natural antioxidative properties via obstructing OS and apoptosis. However, the significant effect of maltol on SCI treatment has never been evaluated yet. In our current study, we explored maltol administration that could trigger the expression of Nrf2 and promote the retranslocation of Nrf2 from the cytosol to the nucleus, which can subsequently obstruct OS signal and apoptosis-mediated neuronal cell death after SCI. Furthermore, we found that maltol treatment enhances PINK1/Parkin-mediated mitophagy in PC12 cells, facilitating the recovery of mitochondrial functions. Our findings propose that maltol could be a promising therapeutic candidate for the treatment and management of SCI.

## 1. Introduction

As a destructive central nervous system injury, spinal cord injury (SCI) leads to a series of motor, sensory, and autonomic nervous dysfunctions. Surprisingly, recent data indicate that about 250,000 to 500,000 patients suffer from SCI worldwide every year, which poses a heavy economic and psychological burden [1, 2]. The pathological process of SCI includes primary injury and secondary injury. The immediate injury is defined as the mechanical injury of the spinal cord. The secondary injuries including inflammation, ischemia, apoptosis, oxidative stress (OS), and mitochondrial dysfunctions further lead to severe tissue damage and inhibit the regeneration of the neuronal nerves in the injured area [3].

The SCI lesion can be accompanied by hypoxia-ischemia and inflammation, causing dysregulation of ion homeostasis, characterized by excessive ROS production, oxidative damage, and neuronal apoptosis. The secondary injury-mediated neuronal apoptosis disrupts neural connection with sensory and motor dysfunctions. Increasing evidence suggests that suppression of the secondary injury-mediated neuronal apoptosis and obstructing the generation of OS deliver a promising therapeutic approach for the treatment and management of SCI [2, 4, 5].

Mitophagy eliminates the damaged mitochondria and plays a pivotal role in maintaining mitochondrial homeostasis and neuronal cell survival [6]. PINK1/Parkin-mediated mitophagy is one of the essential mitophagy pathways [7]. PTEN-induced putative kinase 1 (PINK1) localizes on the outer membrane of the damaged mitochondria; subsequently, recruiting the Parkin protein from the cytoplasm to the mitochondria can activate Parkin protein for its downstream signaling. Parkin is an E3 ubiquitin ligase, which can contribute to the ubiquitination of mitochondrial membrane proteins and eliminate the damaged mitochondrial substances by activating mitophagy [7, 8]. Mitochondrial functions can be improved by scavenging ROS and enhancing mitophagy [9]. Mounting evidence demonstrates that the promotion of mitophagy mitigates neuronal apoptosis and further restores neuronal functions after SCI [6, 10].

Nrf2 pathway activation can suppress OS-induced apoptosis and regulate mitochondrial functions via facilitating mitophagy [11, 12]. Nuclear erythroid 2-related factor 2 (Nrf2) is a crucial regulator against the activation of OS [13]. Under normal circumstances, the activity of Nrf2 is negatively regulated by the formation of Kelch-like ECH-associated protein 1 (Keap1) [14]. Nrf2 is released from Keap1, and the Nrf2 complex further translocates to the nucleus from the cytosol, activating the antioxidant response element (referred to as ARE hereafter). Increasing evidence reports Nrf2 pathway activation triggers multiple genes to express the antioxidant-associated proteins such as heme oxygenase-1 (HO-1), NAD(P)H: quinone oxidoreductase 1 (NQO1), and superoxide dismutase 2 (SOD2) [15, 16].

Accumulating research suggests that some chemical antioxidants such as walnut-derived peptide and MitoQ can enhance mitophagy by obstructing OS and apoptosis via facilitating the Nrf2/PINK1/Parkin signaling pathway [17, 18]. However, maltol (3-hydroxy-2-methyl-4-pyrone, C_6_H_6_O_3_) is a Maillard reaction product by the pyrolysis of starch or sucrose and is widely applied as a reliable food additive and a natural antioxidative agent [19]. Previous studies have shown that maltol intervention could effectively protect peripheral nerves and suppress diabetic-mediated OS [20]. In addition, recent studies have reported that supplementation of maltol can activate the Nrf2/HO-1 signal pathway in the brain by suppressing OS and apoptosis-mediated cell death and alleviating D-Ga-induced neurological impairment l [21]. However, the potential antioxidative effect of maltol in targeting mitophagy and SCI has been elusive.

We performed mouse models and cell lines to evaluate the potential therapeutic effects of maltol on SCI. In our research, we found that maltol treatment could facilitate mitophagy and suppress the activation of OS and neuronal apoptosis via triggering the Nrf2/PINK1/Parkin signaling pathway after SCI. Therefore, our findings suggested that maltol could be a promising therapeutic candidate for the treatment of and management of SCI in the near future.

## 2. Materials and Methods

### 2.1. Animals and Ethics Statement

Sixty adult female 8-week-old C57BL/6 mice (average weight: 20-25 g) were obtained from Wenzhou Medical University, Zhejiang province, China, for conducting our research. Under standard conditions including appropriate temperature (23 ± 2°C), humidity (50 ± 5%), and a 12-hour light/12-hour dark cycle, all animals were placed in the OptiMICE Rotary Experimental Animal Cage System (Catalog No. C89100, Animal Care Systems, USA) and provided with adequate water and foods. All the experimental procedures and protocols were strictly followed according to the guidance for the Animal Care and Use of Laboratory protocols of the National Institutes of Health and finally approved by the Animal Care and Use Committee of Wenzhou Medical University, Zhejiang Province, China.

### 2.2. Contusive SCI Model and Treatment

40 mg/kg pentobarbital sodium 4% (*w*/*v*) was intraperitoneally administered in mice, and laminectomy was performed to remove the part of the vertebra covering the T9-T10 segment of the spinal cord. Then, a 10 g weight steel rod was adopted to damage the exposed spinal cord from 20 mm height, resulting in a moderate contusive SCI model [22, 23]. After the surgical operation, the bladders of the mice were manually emptied twice a day until the bladder's function recovered. All experimental mice were euthanized with excessive pentobarbital sodium on the 3rd, 7th, and 14th days after surgery. The animals were operated on to expose the spinal cord without injury for the sham group. For the SCI + maltol group, maltol (MedChemExpress, USA), 100 mg/kg dose (dissolved in normal saline), was administered to mice one time every day by gavage.

### 2.3. Cell Culture and Treatment

PC12 cells are widely used as an in vitro model to simulate neurons [24]. PC12 cells were purchased from the Cell Storage Center of Wuhan University, Wuhan, China. PC12 cells were carefully cultured and propagated in a suitable environment at 37°C with 5% CO_2_ and 95% air. PC12 cells were cultured in RPMI1640 medium supplemented with 10% fetal bovine serum (FBS, Invitrogen, Carlsbad, CA, USA), 100 U/mL penicillin, and 100 U/mL streptomycin. Control cells were cultured in RPMI1640 complete medium. For the H_2_O_2_ group, H_2_O_2_ (the OS inducer, 200 *μ*M) was incubated to simulate OS for 2 h. To assess the impact of maltol and Nrf2 signal, maltol (2 mM) and the Nrf2 inhibitor ML385 (5 *μ*M, HY-100523, MedChemExpress, USA) were applied for 12 h before H_2_O_2_ treatment.

### 2.4. Locomotion Recovery Assessment

A locomotion recovery assessment was performed at 0, 1, 3, 7, and 14 days (dpi) after SCI, mainly based on the Basso-Mouse-Scale (BMS) score. The BMS scoring ranged from 0 to 9, which primarily evaluates the function of the hind limbs of the experimental mice, consisting of ankle mobility, paw position, coordination, trunk stability, and tail position.

### 2.5. Tissue Preparation

Mice were euthanized at designated time points, and then ventricular perfusion was performed with normal saline solution. The 10 mm-long spinal cord tissues were collected and immediately stored in a refrigerator at -80°C for extracting proteins by western blotting. For histopathological and another staining, the ventricles of mice were perfused with normal saline and 4% paraformaldehyde (PFA). 10 mm-long spinal cords were collected and immediately fixed in a 4% PFA for 48 h. Then, the fixed spinal cords were dehydrated, embedded in paraffin, and cut into 5 *μ*m sections for subsequent staining.

### 2.6. Hematoxylin and Eosin (H&E) and Nissl Stainings

5 *μ*m spinal cord tissue sections were deparaffinized, hydrated, and stained with Hematoxylin-Eosin and Nissl staining commercial kits (Solarbio, China), according to the manufacturer instructions. Finally, the tissue sections were evaluated under a light microscope (Nikon, Japan).

### 2.7. TUNEL Staining

The TUNEL Apoptosis Detection Kit (40307ES20, Yeasen Biochemical) was employed to measure the levels of apoptosis. According to the manufacturer's instructions, TUNEL staining was performed at 7 d after SCI. The section slides (5 *μ*m thick) were adequately deparaffinized and rehydrated. The slides were then carefully stained with a cell death detection kit (Roche, Basel, Switzerland) for 30 min at 37°C. The 4,6-diamidino-2-phenylindole (DAPI) reagent was applied for the nuclei staining. Three independent and blinded observers chose twenty fields of each sample and captured images under a confocal microscope (Nikon, Japan).

### 2.8. Cell Viability Assay

Cell viability was performed by Cell Counting Kit-8 (C0043, Beyotime, China), according to the manufacturer's protocols. PC12 cells were inoculated in 96-well plates (8 × 10^3^ cells per well) and cultured for 24 h. Then, the cells were treated with drugs according to the experimental design. After that, 10 *μ*L of CCK-8 solution was added with a 90 *μ*L basal medium to each well. After 1 h, the absorbance at 450 nm of each sample was measured by a microplate reader.

The Calcein-AM/PI Double Stain Kit (40747ES76, Yeasen, China) is widely performed to detect live cells from dead cells. PC12 cells were incubated with 2 *μ*M Calcein-AM and 4.5 *μ*M PI for 30 min to label live cells and dead cells. Finally, the cells were observed by a confocal microscope (Nikon, Tokyo, Japan).

### 2.9. Intracellular-ROS Generation and MitoSOX Staining

Intracellular-ROS generation was measured by the ROS Assay Kit (S0033, Beyotime, China) and MitoSOX Red Mitochondrial Superoxide Indicator (40778ES50, YEASEN, China), according to the manufacturer's protocols. After being treated with drugs, PC12 cells were incubated with 10 *μ*M DCFH-DA (1 : 1000) for 30 min/5 *μ*M MitoSOX (1 : 1000) in the dark for 40 min at 37°C and washed three times with PBS. The nuclei were then labeled with the DAPI reagent and finally observed by a confocal microscope (Nikon, Tokyo, Japan).

### 2.10. Western Blot Analysis

Spinal cord tissues were collected 3 and 7 days after injury and immediately stored at -80°C for western blot analysis. Spinal tissues and PC12 cells were lysed in ice-cold RIPA buffer solution with 1 mM PMSF (phenyl methane sulfonyl fluoride, Beyotime). To measure the protein concentration of each sample, the BCA protein assay kit (Beyotime) was used according to the manufacturer's protocols. After that, the protein samples were separated via sodium dodecyl sulfate-polyacrylamide (SDS) gel electrophoresis and then carefully transferred to polyvinylidene difluoride membrane (PVDF) (Millipore, USA). The membrane proteins were sufficiently blocked with a 5% skim-milk solution at room temperature for 90 min. Then, the membranes were cut and incubated with specific primary antibodies against Nrf2 (1 : 1000, 16396-1-AP, ProteinTech, USA), HO-1 (1 : 1000, 10701-1-AP, ProteinTech, USA), SOD2 (1 : 2000, 24127-1-AP, ProteinTech, USA), *β*-actin (1 : 1000, 20536-1-AP, ProteinTech, USA), GAPDH (1 : 1000, 10494-1-AP, ProteinTech, USA), Bax (1 : 1000, 50599-2-Ig, ProteinTech, USA), Bcl-2 (1 : 1000, 26593-1-AP, ProteinTech, USA), Histone-H3 (1 : 1000, 17168-1-AP, ProteinTech, USA), LC3 (1 : 1000, 14600-1-AP, ProteinTech, USA), PINK1 (1 : 1000, 23274-1-AP, ProteinTech, USA), Parkin (1 : 1000, 14060-1-AP, ProteinTech, USA), VDAC1 (1 : 1000, 380506, Zen-Bio, China), and TOM20 (1 : 1000, R25952, Zen-Bio, China) for overnight at 4°C. Next, the membranes were incubated with horseradish peroxidase-conjugated secondary (HRP) antibodies (SA00001-1/SA00001-2, ProteinTech, USA) for 90 min at room temperature. The protein bands were carefully evaluated by a ChemiDicTM XRS + Imaging System (Bio-Rad, Berkeley, California, USA). Finally, the bands were quantified using densitometric measurement with the Image Lab 3.0 software.

### 2.11. Immunofluorescence Staining

The spinal cord tissue sections were properly washed in PBS and carefully fixed with 4% paraformaldehyde solution. Then, the tissue slides were carefully permeated in 0.1% Triton X-100 solution for 3 min. Then, the sample slides were adequately blocked with 5% bovine serum albumin (BSA) for 30 min at 37°C. After adequate incubation, the slides were properly washed with PBS solution for 3 times. Then, the slides were incubated with specific primary antibodies including NeuN (1 : 1000, ab104224, Abcam, USA), HO-1 (1 : 300, 10701-1-AP, ProteinTech, USA), Nrf2 (1 : 300, 16396-1-AP, ProteinTech, USA), cleaved caspase-3 (1 : 1000, #9661, CST, USA), and LC3B (1 : 1000, ab192890, Abcam, USA) for overnight at 4°C. MitoTracker (M22426, Thermo Fisher, USA) was diluted with culture medium and then cocultured with living cells for 40 min. Later, Alexa Fluor 488/647-labeled secondary antibodies (1 : 1000, ab150077/ab150115, Abcam, USA) were applied to incubate sections and cells for 60 min at 37°C. Then, the nuclei were stained with the DAPI reagent. The images were finally captured through a confocal laser microscope (Nikon, Tokyo, Japan).

### 2.12. Statistical Analysis

All data were expressed as the mean ± SD. Experiments were repeated at least three times, and the tissues for each triplicate were from different mice. The statistical significances were determined by Student's *t*-test and the one-way analysis of variance (ANOVA) test, followed by Tukey's multiple comparison test. A *P* value < 0.05 was considered a significant value.

## 3. Results

### 3.1. Maltol Reduced Spinal Cord Tissue Damage and Improved Locomotor Function after SCI

**Figure 1:**
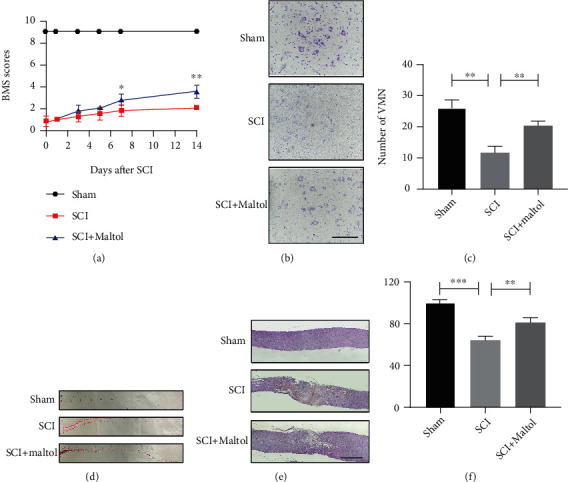
Maltol reduced spinal cord tissue damage and improved locomotor function after SCI. (a) Statistical analysis of BMS in each group from day 1 to day14 after SCI, *n* = 4 per group. (b) Nissl staining sections of the spinal cord at 7 days after SCI. Scale bar: 100 *μ*m (20x). (c) Counting analysis of ventral motor neurons (VMN), *n* = 3 per group. (d) Representative images of footprints in mice in each group at 14 days after SCI. (e) H&E staining sections of the spinal cord at 7 days after SCI. Scale bar: 500 *μ*m. (f) Quantitative analysis of preserved spinal cord tissue percentage, *n* = 3 per group. Values were given as means ± SD. ^∗^*P* < 0.05, ^∗∗^*P* < 0.01, ^∗∗∗^*P* < 0.001.

To determine whether maltol administration alleviates functional recovery after SCI, mice were administered with maltol (100 mg/kg/day) by gavage after surgery. We conducted a 2-week BMS score to assess locomotor function after SCI. The results of the BMS analysis (Figure 1(a)) showed a significant reduction in score after SCI and gradually recovered after a few weeks. In the early stage of SCI (on the 1st and 3rd day), a significant difference between the SCI group and the SCI + maltol group was not noticeably observed, but in the late stage (on the 7th and 14th day), the BMS scores of the maltol-treated SCI model group were remarkably higher than that of the SCI group, indicating that maltol administration could improve locomotor function after SCI. In addition, the representative patterns of the footprints also confirmed this situation (Figure 1(d)).

In addition, H&E and Nissl stainings were applied to observe the destruction of the spinal cord tissue and neuronal loss after SCI. Consistent with motor assessment, Nissl staining and neuronal counting assay indicated that maltol treatment significantly reduced the loss of ventral motor neurons in the SCI + maltol group compared with the SCI group (Figures 1(b) and 1(c)). Moreover, H&E staining showed that maltol administration remarkably reduced peripheral blood and inflammatory cell infiltration, resulting in almost 2-fold reductions in the lesion area after SCI (Figures 1(e) and 1(f)).

### 3.2. Maltol Inhibited OS and Apoptosis Induced by SCI

**Figure 2:**
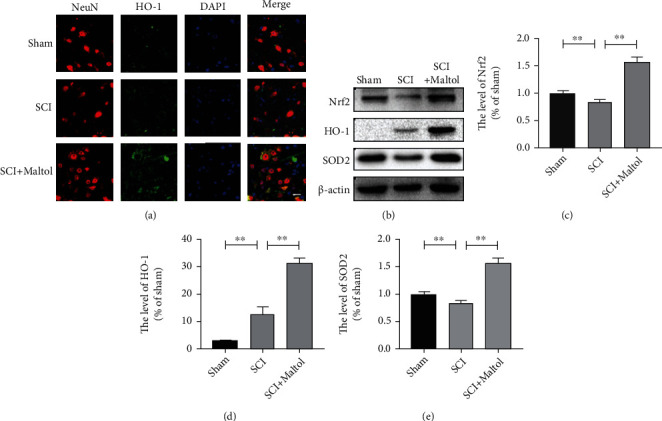
Maltol inhibited oxidative stress caused by SCI. (a) Coimmunofluorescence images show NeuN (red) and HO-1 (green) after SCI. Scale bar = 20 *μ*m. (b–e) Western blots analysis of Nrf2, HO-1, and SOD2. *n* = 3 per group. Values were indicated as means ± SD. ^∗^*P* < 0.05, ^∗∗^*P* < 0.01.

**Figure 3:**
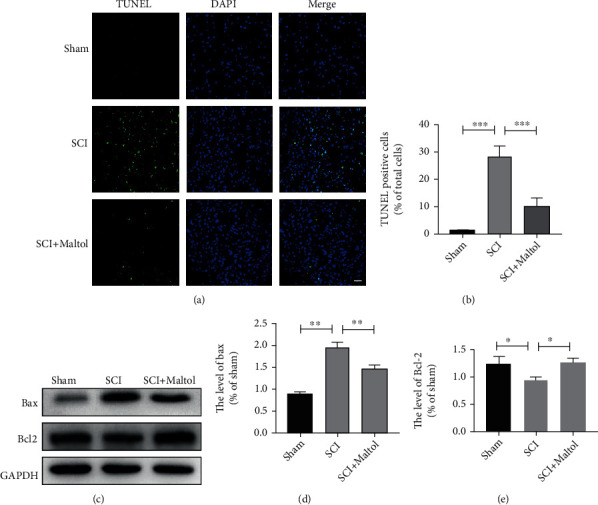
Maltol inhibited apoptosis caused by SCI. (a) TUNEL staining (green) after SCI. Scale bar = 50 *μ*m. (b) Quantitative analysis of TUNEL-positive cells around the injury site, *n* = 3 per group. (c–e) Western blots analysis of Bax and Bcl-2. *n* = 3 per group. Values were given as means ± SD. ^∗^*P* < 0.05, ^∗∗^*P* < 0.01, ^∗∗∗^*P* < 0.001.

Next, we measured the antioxidant and antiapoptotic capacity of maltol administration in SCI. Immunofluorescence findings showed that maltol administration could promote the expression of HO-1 in the neurons, suggesting that maltol improves the antioxidant ability of the neurons (Figure 2(a)). Compared with the nonmaltol mice group, western blot analysis clearly showed that maltol administration could facilitate the expressions of Nrf2 (Figures 2(b) and 2(c)) and the downstream antioxidant-related proteins such as HO-1 and SOD2 (Figures 2(b), 2(d), and 2(e)). TUNEL staining indicated that the level of apoptosis was significantly increased in the SCI mice model. In contrast, maltol administration could remarkably decrease apoptotic levels in mice with SCI (Figures 3(a) and 3(b)). Moreover, western blot analysis exhibited that maltol supplementation could downregulate the expression levels of proapoptotic protein Bax and upregulate the expression levels of antiapoptotic protein Bcl-2 in the injured mice model (Figures 3(c)–3(e)). Thus, these above findings suggested that maltol could restrict the activation of OS- and SCI-induced neuronal apoptosis.

### 3.3. Maltol Attenuated H_2_O_2_-Induced OS and Apoptosis in PC12 Cells

**Figure 4:**
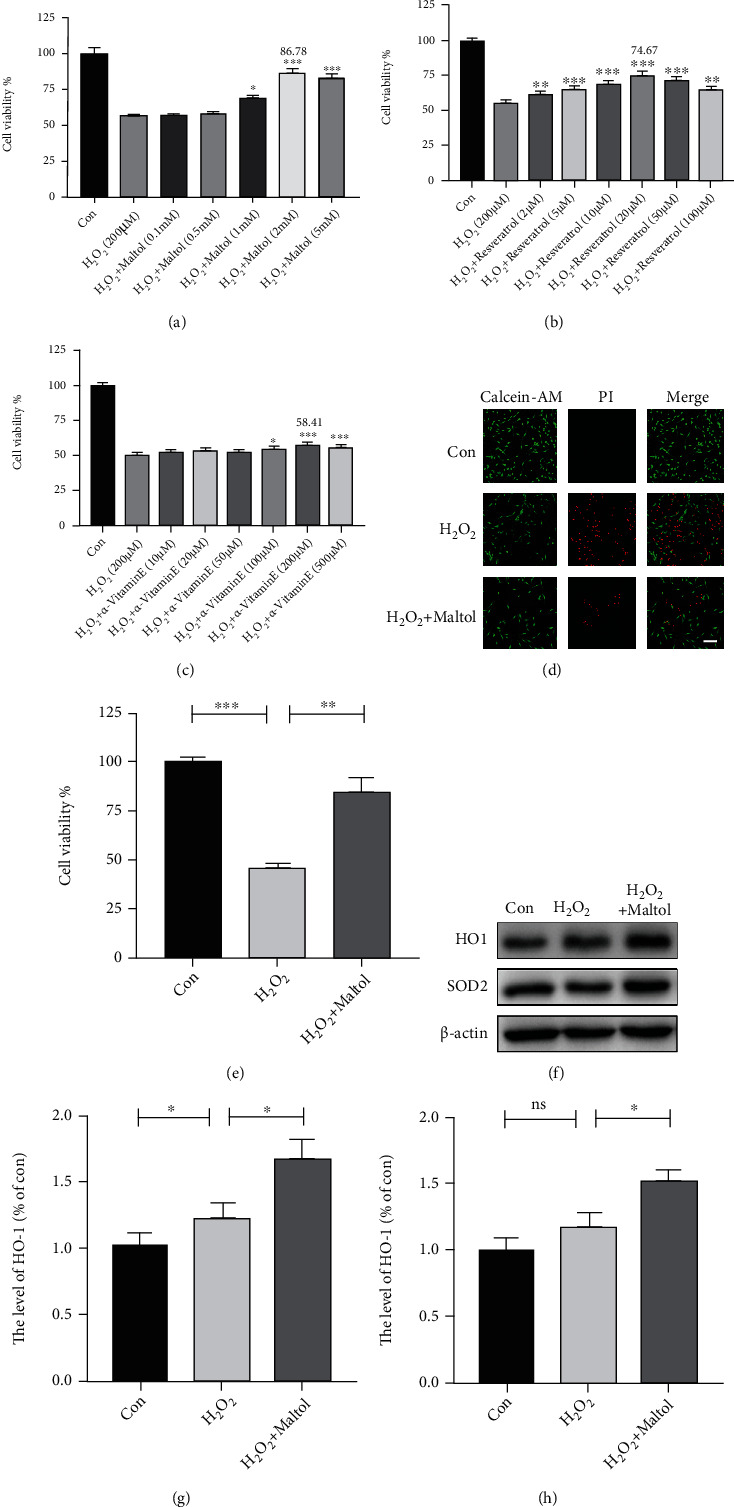
Maltol attenuated H_2_O_2_-induced oxidative stress and apoptosis in PC12 cells. (a–c) Adopting CCK-8 to measure relative cell viability after being treated with maltol, resveratrol, and *α*-vitamin E in different concentrations. *n* = 4 per group. ^∗^*P* < 0.05, ^∗∗^*P* < 0.01, ^∗∗∗^*P* < 0.001 vs. H_2_O_2_ group. (d–e) Adopting the Calcein-AM/PI Double Stain Kit to distinguish live cells (green) from dead cells (red) and calculate cell viability. Scale bar = 100 *μ*m. (f–h) Western blots analysis of HO-1 and SOD2 in vitro experiments. *n* = 3 per group. Values were given as means ± SD. ^∗^*P* < 0.05, ^∗∗^*P* < 0.01, ^∗∗∗^*P* < 0.001, and NS (not significant).

We then investigated whether maltol could alleviate OS and apoptosis-mediated neuronal cell death in vitro experiment. PC12 cells are generally employed to simulate neurons in the central nervous system [24]. H_2_O_2_ is widely used to induce OS in cells. Our investigation found that the cell viability treated with maltol was positively correlated with the dose of maltol within a specific concentration range (0.1-2 mM). Cell viability assay revealed that maltol treatment at a dose-dependent manner could enhance cell viability rate in PC12 cells (Figure 4(a)).

Interestingly, compared to other natural antioxidants, resveratrol and *α*-vitamin E, maltol treatment could significantly suppress OS-induced apoptosis. Primarily, compared with *α*-vitamin E, the cell viability with maltol treatment increased about 30% (Figures 4(a)–4(c)). The Calcein-AM/PI Double Stain results also confirmed that maltol treatment could reduce the apoptosis of PC12 cells (Figures 4(d) and 4(e)). Our findings demonstrated that maltol could significantly inhibit H_2_O_2_-induced apoptosis. We further speculated that maltol upregulated the expression level of antioxidant proteins. Our western blot analysis revealed that maltol could promote the expressions of antioxidant-related proteins such as HO-1 and SOD2 (Figures 4(f)–4(h)).

### 3.4. Maltol Attenuated OS and Apoptosis by Activating the Nrf2/ARE Signaling Pathway in PC12 Cells

**Figure 5:**
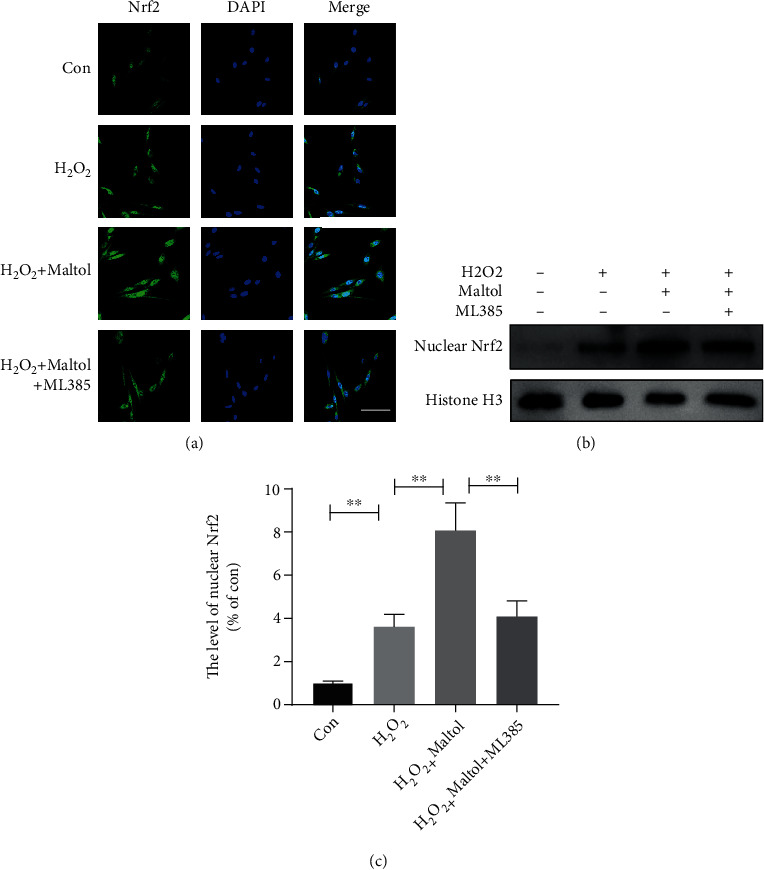
Maltol activated the Nrf2 signaling pathway in PC12 cells. (a) Immunofluorescence staining of Nrf2 (green) in PC12 cells. Scale bar = 50 *μ*m. (b, c) Western blots analysis of Nrf2 in the nucleus. *n* = 3 per group. Values were given as means ± SD. ^∗∗^*P* < 0.01.

We further explored whether maltol treatment could promote the Nrf2 signaling pathway and obstruct the activation of OS and apoptosis-mediated cell death in PC12 cells. As shown by the immunofluorescence staining, maltol treatment increased the fluorescence intensity of Nrf2 and promoted the retranslocation of Nrf2 from the cytosol to the nucleus (Figure 5(a)). The application of ML385, a specific inhibitor of Nrf2, could reverse this potential effect. Meanwhile, the western blot assay also showed that maltol administration could facilitate the expression of Nrf2 in the nucleus (Figures 5(b) and 5(c)). Therefore, our findings suggested that maltol might significantly trigger the Nrf2 signaling pathway.

**Figure 6:**
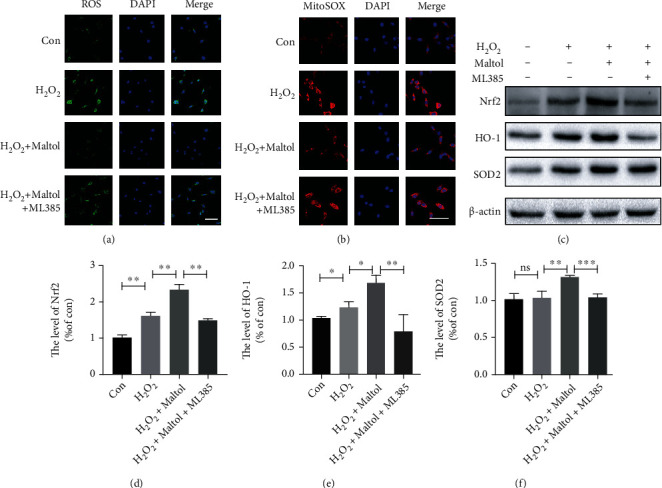
Maltol attenuated oxidative stress by activating the Nrf2/ARE pathway in PC12 cells. (a) Detection of ROS (green) production by the ROS Assay Kit in PC12 cells. Scale bar = 50 *μ*m. (b) MitoSOX staining (red) in PC12 cells. Scale bar = 50 *μ*m. (c–f) Western blots analysis of Nrf2, HO-1, and SOD2. *n* = 3 per group. Values were given as means ± SD. ^∗^*P* < 0.05, ^∗∗^*P* < 0.01, ^∗∗∗^*P* < 0.001, and NS (not significant).

Next, we illustrated the connections between intracellular OS and the Nrf2 signaling pathway. We employed that 2′,7′-dichlorodihydrofluorescein diacetate (DCFH-DA) can be oxidized into fluorescent DCF by ROS, to assess ROS formation (Figure 6(a)). Our results showed that maltol treatment could dramatically decrease ROS formation in H_2_O_2_-stimulated PC12 cells. Significantly, ML385 reversed the inhibitory effect of maltol on ROS formation. Furthermore, we applied MitoSOX to detect mitochondrial superoxide and maltol could significantly restrict mitochondrial OS generation, indicating maltol delivers a protective effect on mitochondrial damage (Figure 6(b)). The western blot analysis exhibited that maltol could promote Nrf2, HO-1, and SOD2 in PC12 cells, while the application of ML385 decreased the expression of these antioxidant-related proteins (Figures 6(c)–6(f)). Therefore, maltol intervention could reduce ROS production and improve antioxidant capacity by enhancing the Nrf2 signaling pathway, which is consistent with the previous research [14].

**Figure 7:**
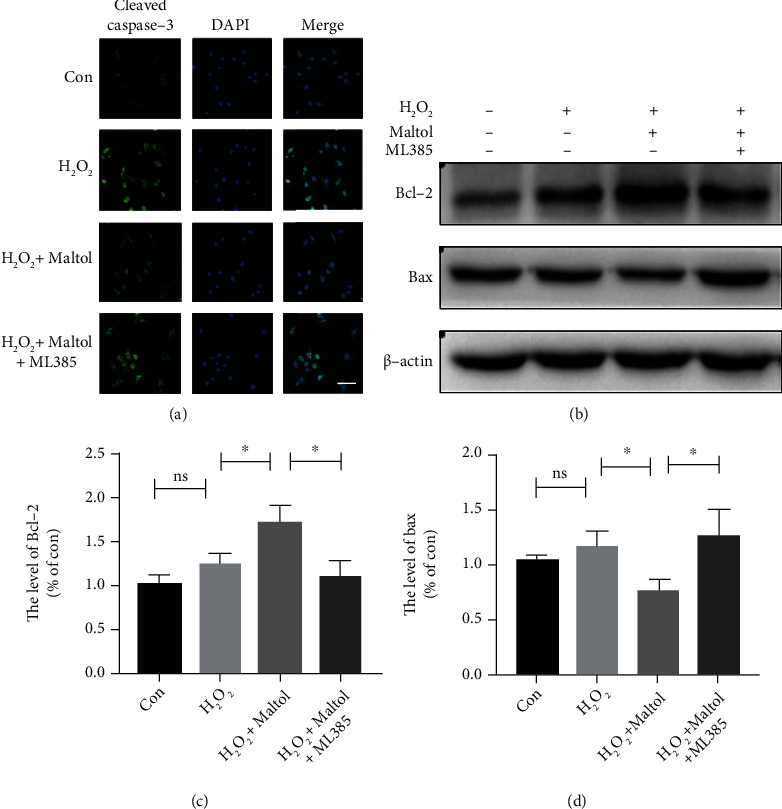
Maltol attenuated apoptosis by activating the Nrf2/ARE pathway in PC12 cells. (a) Immunofluorescence staining of cleaved caspase-3 (green) in PC12 cells. Scale bar = 50 *μ*m. (b–d) Western blots analysis of Bax and Bcl-2. *n* = 3 per group. Values were given as means ± SD. ^∗^*P* < 0.05 and NS (not significant).

We aimed to determine whether maltol could suppress apoptosis-mediated neuronal cell death via promoting the Nrf2 signaling pathway in PC12 cells. Cleaved caspase-3 is widely applied to evaluate the level of apoptosis. Our immunofluorescence findings displayed that maltol treatment could remarkably reduce the fluorescence intensity of cleaved caspase-3. Furthermore, the application of ML385 could reverse the antiapoptotic effect of Nrf2, which further supported that Nrf2 suppressed apoptosis via enhancing its antioxidative effects (Figure 7(a)). To further evaluate the antiapoptosis effect of maltol, we performed the western blot assay to determine the expression levels of the antiapoptotic protein Bcl-2 and the proapoptotic protein Bax. Consistently, maltol treatment could downregulate the expression level of Bax and upregulate the expression level of Bcl-2 in PC12 cells, but ML385 reversely regulated the expression patterns of these two proteins (Figures 7(b)–7(d)). In conclusion, these findings suggested that maltol could effectively suppress apoptosis via enhancing the Nrf2 signaling pathway.

### 3.5. Maltol Increased the Level of Mitophagy by Activating the Nrf2/PINK1/Parkin Signaling Pathway in PC12 Cells

**Figure 8:**
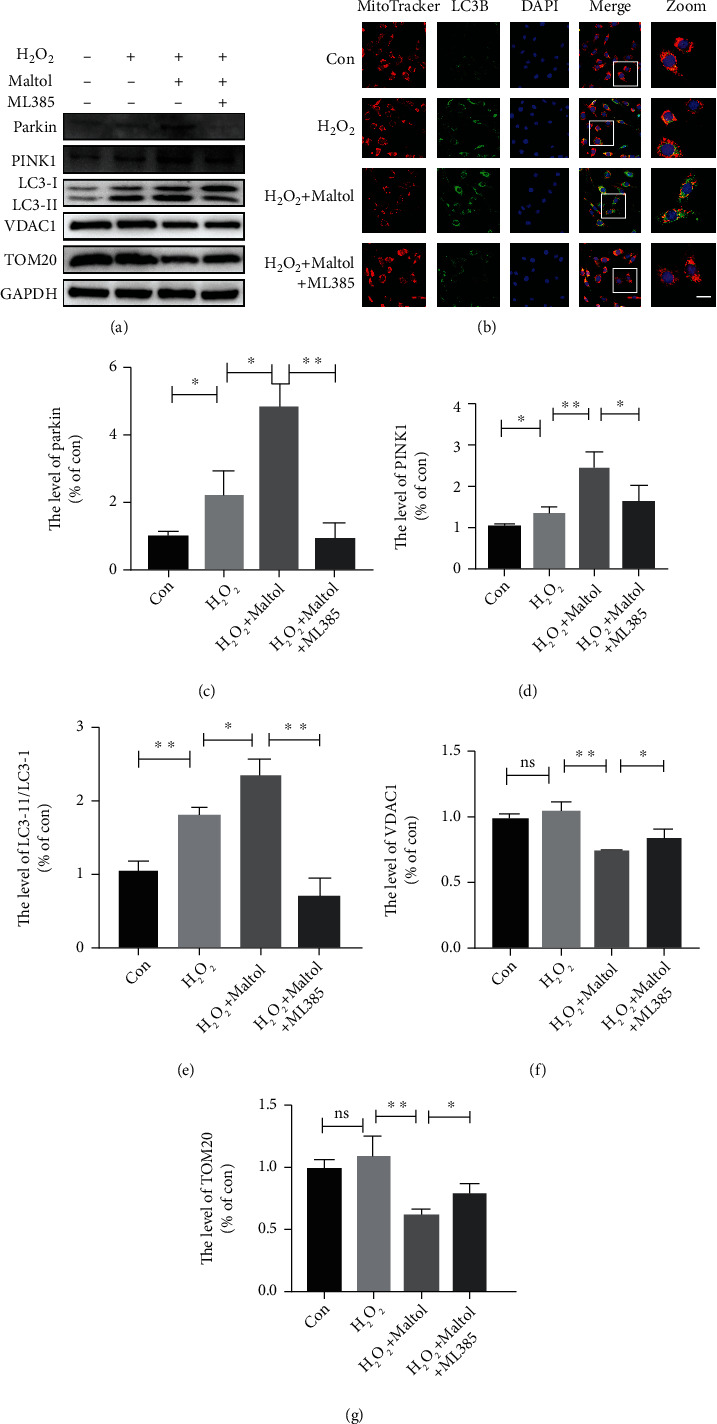
Maltol increased the level of mitophagy by activating the Nrf2/PINK1 signaling pathway in PC12 cells. (a, c–g) Western blots analysis of PINK1, Parkin, LC3, VDAC1, and TOM20. *n* = 3 per group. (b) Coimmunofluorescence images show MitoTracker (red) and LC3B (green). Scale bar = 10 *μ*m. Values were given as means ± SD. ^∗^*P* < 0.05, ^∗∗^*P* < 0.01, and NS (not significant).

We explored whether Nrf2 signaling pathway activation could facilitate mitophagy. Therefore, we first determined the expression levels of two well-known mitophagy-associated proteins such as PINK1 and Parkin. Western blot outcomes clearly demonstrated that the expression levels of these two proteins were dramatically upregulated in PC12 cells (Figures 8(a), 8(c), and 8(d)). Meanwhile, maltol treatment could reduce mitochondrial membrane proteins VDAC1 and TOM20 (Figures 8(a), 8(f), and 8(g)). Immunofluorescence findings showed reductions in the numbers of mitochondria (Figure 8(b)), indicating the activation of mitophagy. To confirm the event of mitophagy, we performed a confocal microscopic examination to ascertain the colocalization of LC3B (autophagosome) and MitoTracker (mitochondria). Surprisingly, immunofluorescence findings revealed that maltol treatment increased colocalization of LC3B with MitoTracker. Meanwhile, the application of ML385 in PC12 cells led to the mislocalization of LC3B and MitoTracker (Figure 8(b)). Furthermore, maltol treatment could increase the expression of LC3, and ML385 could inhibit the expression level of LC3 (Figures 8(a) and 8(e)).

## 4. Discussion

SCI is a severe central nervous system injury, increasing year by year with a tendency toward young people. The development of effective treatment has always been a significant challenge in neuroprotection and nerve regeneration after SCI [2, 25, 26]. The severe secondary damage caused by SCI including ischemia and hypoxia and inflammatory edema induces excessive ROS production, which eventually facilitates massive apoptosis-mediated neuronal cell death in the centre of the injury and surrounding areas, resulting in severe motor and sensory damage [27, 28]. Therefore, it is an urgent need to develop effective drugs to treat and manage SCI.

**Figure 9:**
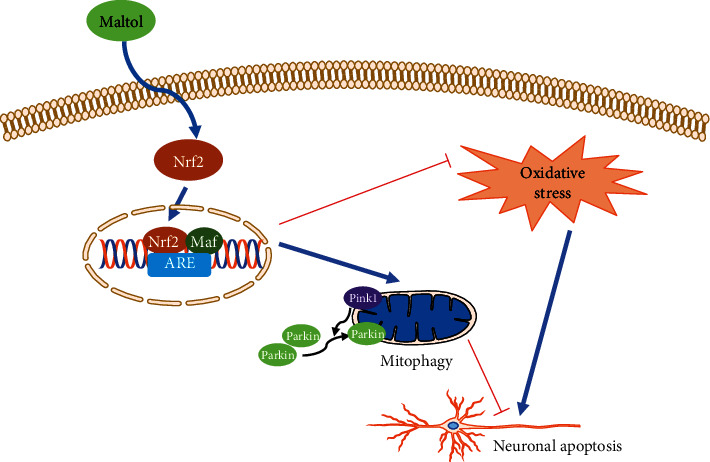
The potential molecular mechanism of maltol on SCI. Maltol can activate the expression of Nrf2 and facilitate Nrf2's translocation from the cytosol to the nuclear. Then it promotes mitophagy via enhancing the Nrf2/PINK1/Parkin pathway, ultimately suppressing the neuronal apoptosis in SCI.

Maltol, a product of the starch and sucrose pyrolysis process formed by the Maillard reaction, is widely used as a natural antioxidant [29, 30]. Numerous experiments have reported that maltol exerts significant antioxidant and antiapoptotic effects in treating and managing multiple diseases. In addition, a previous study revealed that maltol intervention promoted the recovery of peripheral nerves and alleviated diabetic peripheral neuropathy through suppressing OS and apoptosis [20]. Another study conducted by Liu and colleagues confirmed that maltol could facilitate the activities of glutathione (GSH) and superoxide dismutase (SOD) in the liver, promote HO-1 expression, and obstruct carbon tetrachloride-induced apoptosis [31]. However, no relevant studies have been reported on the protective effects of maltol and its specific mechanism to alleviate the progression of secondary SCI. Therefore, we aimed to investigate the protective mechanism of maltol in the treatment of SCI. To assess the significant effects of maltol in inhibiting the activation of OS, we compared maltol with other natural antioxidants. Compared with resveratrol and vitamin E, maltol could significantly suppress OS-driven apoptosis. Our present study further confirmed that maltol activated the Nrf2 signal and promoted PINK1/Parkin-mediated mitophagy to improve mitochondrial functions (Figure 9).

After SCI, spinal cord ischemia is accompanied by subsequent energy exhaustion and ATP deficiency, increasing intracellular calcium concentration and ultimately overproduction of ROS [32]. Excessive ROS formation increases the oxidative damage of proteins, DNA, and lipids molecules and promotes apoptosis-mediated cell death [33]. Our results suggested that gavage of maltol significantly promoted the expression of antioxidant-related proteins HO-1 and SOD2 in the SCI mouse models. Furthermore, our immunofluorescence assay revealed that maltol supplementation could enhance the antioxidant capacity of neurons in the injured spinal cord tissue area. Meanwhile, we exposed PC12 cells to H_2_O_2_ to induce OS and treated them with maltol. Our results indicated that maltol could obstruct ROS and MitoSOX production in PC12 cells. Interestingly, our findings uncovered that inhibition of OS significantly attenuated neuronal apoptosis. In addition, our results demonstrated that oral administration of maltol significantly promoted the survival of neurons in mice and the survival of PC12 cells in vitro.

Nrf2 is a crucial transcription factor in the regulation of antioxidative stress. After binding to ARE in the nucleus, Nrf2 signals can initiate the expressions of a variety of antioxidant, anti-inflammatory, and antiapoptosis genes [34, 35]. Previous studies have shown that maltol activates the Nrf2 signaling pathway. Interestingly, Sha and coworkers demonstrated that maltol supplementation could reduce the levels of OS and restain apoptosis via promoting the Nrf2/HO-1 signaling axis in the brain tissue alleviating D-Gal-induced neurological impairment [21]. A pioneering research reports that maltol administration attenuates the aggravation of osteoarthritis via enhancing the Nrf2/HO-1 signaling pathway [36]. Our study is consistent with the above analysis that maltol can activate the Nrf2 signal pathway. Our findings revealed that maltol could promote the expression of Nrf2 in the spinal cord after SCI. In PC12 cells, maltol could dramatically boost the translocation of Nrf2 into the nucleus. Then the activated Nrf2 fosters the expression of HO-1 and SOD2, which was further confirmed by the application of the Nrf2 inhibitor.

Mitophagy is a process of the selective removal of damaged and senescent mitochondria and regulates mitochondrial quality and homeostasis [37]. Growing evidence indicates that PINK1/Parkin is a classical mitochondrial autophagic pathway [8, 38, 39]. During mitochondrial damage or ageing, the mitochondrial membrane potential decreases, and PINK1 accumulates in the outer membrane and promotes the activation and translocation of Parkin, which eventually leads to the degradation of mitochondrial proteins such as TOM20 and VDAC1 [40]. Emerging evidence revealed that Nrf2, a transcription factor, regulates various cytoprotective genes and mitochondrial functions [41]. Nrf2 signaling pathway activation regulates membrane potential activity, antioxidant defense, biogenesis, and integrity in mitochondria [11, 42]. Recent literature reports that Nrf2 can remarkably trigger mitophagy through the Nrf2/PINK1 signaling axis. Interestingly, Bertrand and colleagues found that the synthetic Nrf2 activator, 1,4-diphenyl-1,2,3-triazole compound, promotes mitophagy and plays a contributory role in regulating mitochondrial homeostasis [43]. Current studies have found that some potential antioxidants such as walnut-derived peptide and MitoQ can facilitate PINK1/Parkin-mediated mitophagy and obstruct OS by activating the Nrf2 signaling pathway [17, 18]. However, the potential targets of maltol on mitochondrial functions have not been well studied. Thus, we speculated that maltol could facilitate the Nrf2/PINK1/Parkin signaling pathway in SCI mouse models. Our results confirmed that maltol administration could trigger the Nrf2 pathway, which further promoted the expression of PINK1 and Parkin in PC12 cells.

Interestingly, maltol treatment could reduce the expression levels of mitochondrial membrane proteins including TOM20 and VDAC1 in PC12 cells. Immunofluorescence findings clearly indicated that maltol could significantly facilitate mitochondrial function and autophagosome colocalization. In a word, our results strongly suggested that maltol administration could promote mitophagy in the SCI mice model. Our investigation also revealed that the application of Nrf2 inhibitor reversed the above effect.

Indeed, there are still some limitations in our current study. For instance, other aspects of mitochondrial functions including mitochondrial membrane potential and mitochondrial biogenesis have not been further investigated. More animal experiments might be needed to further prove the results of our cell experiments. In addition, the evaluation of the long-term effects of maltol intervention on SCI is also unknown in our current investigation. Further studies are highly warranted to explore the long-term pharmacological effects of maltol intervention in SCI. Mechanistically, our research found that maltol intervention could significantly promote mitophagy and obstruct OS signals and apoptosis-mediated neuronal cell death through enhancing the Nrf2/PINK1/Parkin pathway after SCI, suggesting that maltol could be a promising therapeutic agent for the treatment and management of SCI.

## Data Availability

The original data supporting the conclusions of this article can be available from the corresponding authors upon request.
